# The role of synovial mesenchymal stem cell-derived exosomes in cartilage repair: a systematic review

**DOI:** 10.3389/fphar.2025.1617874

**Published:** 2025-06-25

**Authors:** Jiemao Su, Yansong Qi, Lin Niu, Yongxiang Wang, Baogang Wei, Bingxian Ma, Keyu Kong, Zanjing Zhai, Yongsheng Xu

**Affiliations:** ^1^ Orthopedic Center (Sport Medicine Center), Inner Mongolia Autonomous Region People’s Hospital, Hohhot, China; ^2^ Shanghai Key Laboratory of Orthopaedic Implants, Department of Orthopaedic Surgery Shanghai Ninth People’s Hospital, Shanghai Jiaotong University School of Medicine, Shanghai, China

**Keywords:** synovial, mesenchymal stem cells, exosomes, cartilage, knee, osteoarthritis

## Abstract

**Background:**

Knee osteoarthritis (KOA) is the most widespread degenerative disease in the cumulative population. With the increasing aging of the population, KOA has become one of the most important factors leading to joint deformities in middle-aged and elderly people. At present, the therapeutic effect of synovial mesenchymal stem cells (SMSCs) has gradually attracted the attention of many researchers. Due to their better chondrogenic ability, they have gradually become an effective way to treat cartilage injury. Because its function mainly relies on exosomes and exosomes have many advantages of cell-free therapy, it has attracted much attention from researchers.

**Methods:**

The study was searched between April 20, 2014, and April 20, 2025, on China National Knowledge Infrastructure (CNKI), Wanfang database, PubMed, the Cochrane Library, and Web of Science. Two researchers independently reviewed the literature, extracted data, evaluated bias. In cases of disagreement, a third reviewer made the final decision.

**Results:**

The initial literature search identified 198 potentially relevant studies. After removing 7 duplicate publications, 183 records remained for screening. Title and abstract review excluded 164 irrelevant studies. Full-text assessment was performed on the remaining 19 articles, of which 12 ultimately qualified for inclusion. Overall, the risk of bias in most of the eligible studies was unclear. In the 12 included studies, it was confirmed that SMSC-derived exosomes could maintain and promote cartilage repair and reduce the degree of cartilage damage by in vitro cell experiments. By isolating and extracting the main functional mirnas, it was found that these functional mirnas had a good therapeutic effect on cartilage injury.

**Conclusion:**

SMSC-derived exosomes demonstrate significant potential for cartilage repair in KOA, primarily mediated by functional miRNAs. While in vitro results are promising, the unclear risk of bias in current studies underscores the need for higher-quality clinical research to validate their therapeutic application.

**Systematic Review Registration:**

identifier [CRD420250651715].

## 1 Introduction

Osteoarthritis (OA) is the most common joint disease, affecting over 500 million individuals globally, with more than half of these cases involving knee OA (KOA) (Hunter et al., 2020). Recent research has increasingly focused on the role of systemic risk factors in KOA development. Notably, population aging and the global obesity epidemic have significantly contributed to the rising prevalence of KOA (Wallace et al., 2017). Additionally, sedentary lifestyles, comorbid metabolic syndrome, and increased reliance on pain medications further exacerbate KOA progression (Davis et al., 2019; Kaufman et al., 2012; Roos and Arden, 2016; Zhuo et al., 2012). As a multifactorial condition influenced by both local and systemic factors, its precise etiology remains unclear, and no curative treatment is currently available (Michael et al., 2010). At present, the most effective treatment methods for KOA are artificial joint replacement and osteotomy, but they are all end-stage treatments (Ramalho et al., 2025). In the current social situation, the economic burden of surgical treatment for patients has been greatly improved, but the risk of injury and complications caused by surgery is still inevitable. Therefore, experts and scholars suggest that the treatment threshold should be moved forward and treated in the early and middle stages of KOA in order to delay the progress of KOA (Diab et al., 2025).

Clinicians and researchers are now focusing on the treatment of cartilage damage in the early and middle stages of KOA. Cartilage injury is one of the primary causes of KOA and a major contributor to knee pain. When cartilage is damaged, the degeneration of articular cartilage accelerates, leading to pain and restricted movement in patients. Therefore, targeted treatment of damaged articular cartilage is critica (Bernhard and Vunjak-Novakovic, 2016; Kanamiya et al., 2002; Lyu et al., 2022). Currently, various therapeutic options are available for cartilage injury, including microfracture, allograft cartilage transplantation, and xenograft cartilage transplantation. However, these methods still require surgery, and the above manipulations are performed during the operation.

Among the current non-surgical treatment options, Platelet-Rich Plasma (PRP) is favored by many patients and physicians because it is collected from their own blood, the body will not produce rejection, and has small trauma and good efficacy (Yan et al., 2025). PRP treatment can produce good curative effect mainly because it contains many growth factors, which can accelerate the proliferation of chondrocytes and cartilage tissue repair (Everts et al., 2020; Rodríguez-Merchán, 2022). Cell therapy is the direct injection of cells with differentiation ability such as adipose-derived mesenchymal stem cells into the joint cavity to achieve chondrocyte regeneration.

MSC, as the regenerative agent, is one of the treatments for knee OA treatment due to its potential to heal cartilage defects (Sergijenko et al., 2016). A large number of studies have shown that the stimulation of bone marrow MSCs (Wang et al., 2024), adipose MSCs (Ruan et al., 2024), synovial MSC (Gao et al., 2024), and umbilical cord MSCs (Najar et al., 2024) will accelerate their differentiation into chondrocytes and has good therapeutic effects on KOA-induced cartilage defects. In addition to the function of chondrocytes, mesenchymal stem cells also show strong regulatory ability and achieve immune regulation by improving local microenvironment (Lee et al., 2015; Li N. et al., 2020), which can be inhibited by chronic inflammation. However, comparative studies have determined that synovial MSCs (SMSCs) have more chondrogenic potential than MSCs from other tissues (Sekiya et al., 2015; Tjandra et al., 2024; Futami et al., 2012), because they are committed to a chondrogenic lineage (Koyama et al., 2008).

Mesenchymal stem cells can achieve the above functions mainly through extracellular vesicles. Extracellular vesicles, as mediators of information transmission between cells, play an extremely important role in communication in cell differentiation and regulation of the microenvironment.

### 1.1 Anatomy and physiological basis of the SMSC-Exos

**FIGURE 1:**
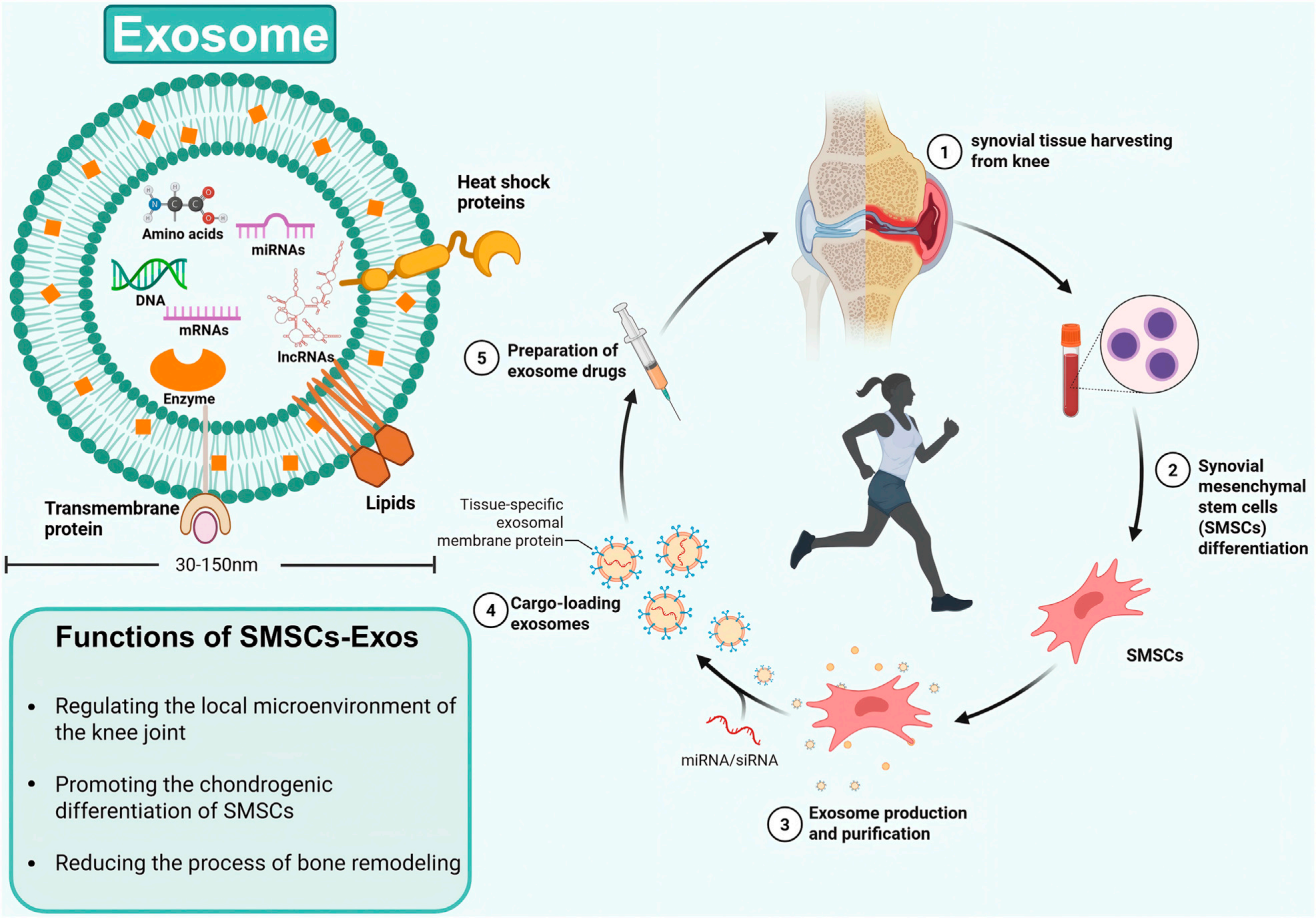
The structure and function of SMSC-derived exosomes, as well as their extraction and treatment processes.

Exosomes are membrane-bound vesicles with a diameter ranging from 30 nm to 150 nm and are found in almost all living organisms (Gross et al., 2012; Mathivanan et al., 2010; Simons and Raposo, 2009). In addition to proteins, exosomes contain various nucleic acids, including mRNAs, microRNAs (miRNAs), and other non-coding RNAs (ncRNAs) (Sato-Kuwabara et al., 2015). As intercellular messengers, exosomes contribute to the healing of osteoarthritic cartilage via paracrine mechanisms (Kim et al., 2020). An increasing number of studies have demonstrated that exosomes derived from mesenchymal stem cells can carry a variety of biomolecules and mediate intercellular communication (Jo et al., 2023), thereby achieving therapeutic effects similar to those of their parental cells (Rani et al., 2015). Exosomes derived from mesenchymal stem cells may offer distinct advantages over whole-cell treatments in terms of patient safety, such as reduced immunoreactivity and no risk of tumor formation (Hassanzadeh et al., 2021). Synovium, a specialized connective tissue lining the inner wall of joints, contains abundant macrophages and fibroblastic cells (Haubruck et al., 2021), these cells are involved in tissue repair under the regulation of SMSCS when the microenvironment of the knee joint changes (Figure 1).

**FIGURE 2:**
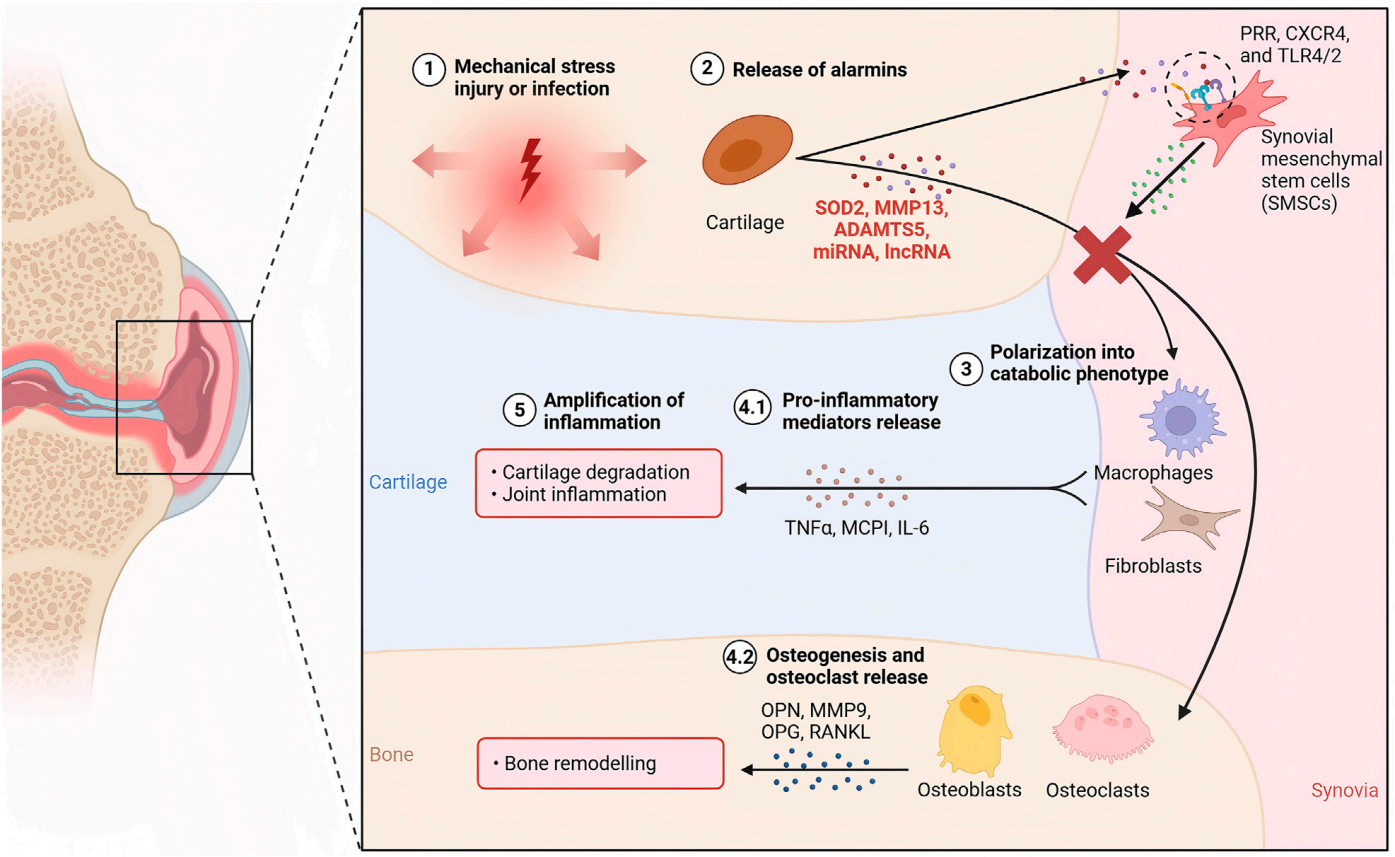
The mechanism of KOA cartilage injury and the intervention mechanism of SMSC-derived exosomes.

SMSCs represent a specialized population of multipotent stromal cells residing within the synovial tissue of the knee joint. These cells exhibit trilineage differentiation potential, demonstrating the capacity to undergo osteogenic, adipogenic, and chondrogenic differentiation under appropriate physiological or pathological conditions (Sakaguchi et al., 2005). Functionally, SMSCs play a crucial role in maintaining joint homeostasis through their paracrine activities, particularly via the secretion of exosomes. These membrane-bound vesicles serve as important mediators of intercellular communication, facilitating the transfer of bioactive molecules such as proteins, lipids, and nucleic acids to recipient cells (Lu M. et al., 2025). Through this exosome-mediated signaling mechanism, SMSCs actively participate in the regulation of the local joint microenvironment, influencing cellular behaviors including proliferation, migration, and importantly, the lineage commitment of neighboring stem cells (Yang D. et al., 2025). The precise composition of these exosomal cargos, which varies according to physiological demands and pathological states, determines the specific differentiation pathways induced in target SMSCs, thereby contributing to joint tissue maintenance and repair processes. Exosomes isolated from osteoarthritic synovial fluid have been shown to inhibit the progression of osteoarthritis (Headland et al., 2015) (Figure 2).

### 1.2 Anatomy and physiological basis of the knee cartilage

The articulating end of joint bones is covered by articular cartilage, which has a thickness of 1–4 mm (Shepherd and Seedhom, 1999), and functions to transmit joint loads while maintaining a low frictional coefficient. Articular cartilage is a highly specialized connective tissue composed of sparsely distributed chondrocytes (<2%–5% by volume of articular cartilage (Muir, 1995)) surrounded by a dense extracellular matrix (ECM). Water, either bound (water of hydration) or unbound, is the most abundant component, accounting for approximately 70% of the wet weight in healthy articular cartilage. The water content increases from 65% near the subchondral bone to 80% in the superficial zone (Amadò et al., 1976). Due to the avascular nature of cartilage, water within the tissue plays a critical role in nutrient transport to chondrocytes (Maroudas, 1970). In addition to water, cartilage contains a variety of proteins, macromolecules, and lipids (Seror et al., 2015). Healthy articular cartilage exhibits one of the most efficient lubrication systems in nature, with friction coefficients as low as 0.001 under physiologically high pressures (Lin and Klein, 2021).

### 1.3 Current treatment of the knee cartilage damage

At present, there are a variety of treatment options for articular cartilage injury, all of which have shown beneficial therapeutic results but also have associated negative effects. Allogeneic cartilage microparticle transplantation is the ideal approach for small-area cartilage lesions, particularly those less than 2 mm^2^ (Lin et al., 2010). However, this method will ultimately result in difficulties such as subchondral bone collapse, poor cartilage fixation at the transplant site, and poor development of the transplanted and surrounding cartilage. Microfracture is the most common treatment. Microfracture involves drilling holes in the cartilage until bone marrow can be seen oozing out. A recent study on microfracture showed that, while it is effective in filling cartilage defects, it is ineffective in improving clinical symptoms (Lee et al., 2019). This method causes the production of fibrocartilage, which differ from the functional morphology of the hyaline cartilage (Li et al., 2022), there is no hyaline cartilage to reduce friction.

Autologous cultured chondrocytes are the most commonly employed cell-based approach for cartilage defect repair. However, during *in vitro* expansion, these cells frequently undergo phenotypic dedifferentiation, leading to the formation of fibrocartilage—rather than the desired hyaline cartilage—in the regenerated tissue (Brittberg et al., 1994; Huey et al., 2012; Roberts et al., 2009). Currently, allogeneic cartilage micrografts have emerged as a leading treatment for cartilage defects. Animal studies demonstrate that this approach significantly improves International Knee Documentation Committee (IKDC) scores while markedly reducing pain levels, as assessed by the Visual Analog Scale (VAS) (Farr and Yao, 2011). This approach not only minimizes surgical trauma—enhancing patient acceptability—but also optimally stimulates cartilage regeneration. Although the above treatment is effective, surgery is still needed, and some patients cannot cooperate well with the doctors due to the fear of surgery. Therefore, non-surgical treatment has gradually become the main breakthrough point for the treatment of cartilage injury in the future.

As an intra-articular injection therapy without surgery, cell therapy has been favored by many patients. Mesenchymal stem cells can secrete a variety of growth factors and stimulating factors to promote chondrocyte regeneration and cartilage tissue repair (Yang H. et al., 2025). Moreover, under the influence of the inflammatory microenvironment in the knee joint, mesenchymal stem cells can differentiate into chondrocytes to achieve the purpose of cartilage regeneration. However, cell therapy also has many drawbacks. The differentiation direction of MSC is uncertain. MSC can differentiate into bone, cartilage and fat, and its differentiation direction is difficult to control *in vivo* (Lu J. S. et al., 2025). The storage and transport conditions of MSC are strict, and it is difficult to achieve storage and transport while maintaining cell activity. After topical application, it will produce immunogenicity, stimulate the local rejection reaction and aggravate the patient’s symptoms (Lu J. S. et al., 2025).

As a cell-free therapy, extracellular vesicles secreted by MSC avoid the defects caused by cell therapy and have the therapeutic advantages of MSC, which has become the object of in-depth research by many researchers. Knee synovial mesenchymal stem cells are terminal stem cells, and their differentiation direction is more likely to induce cartilage (Aghajani et al., 2025). Therefore, SMSC-derived exosomes also play a role in promoting cartilage repair. However, the mechanism of SMSC-derived exosomes in the treatment of cartilage injury has not been fully elucidated. This study conducted a systematic literature review to assess the therapeutic efficacy of SMSC-derived exosomes for knee joint cartilage repair. We hypothesized that exosome-based intervention would demonstrate significant clinical improvement.

## 2 Methods

This systematic review was performed in strict accordance with: guidelines for preclinical systematic reviews and meta-analyses of animal studies (Neeleman et al., 2024), and The Preferred Reporting Items for Systematic Reviews and Meta-Analyses (PRISMA) statement (Bruzek et al., 2024). To ensure originality, we conducted a preliminary search of the PROSPERO database (registration number: CRD420250651715) to identify and avoid overlap with ongoing systematic reviews).

### 2.1 Search strategy

This systematic review was conducted according to PRISMA (Preferred Reporting Items for Systematic Reviews and Meta-Analyses) criteria, along with a PRISMA checklist. Each study was searched between 20 April 2014, and 20 April 2025, on China National Knowledge Infrastructure (CNKI), Wanfang database, PubMed, the Cochrane Library, and Web of Science. The electronic search strategy used was “SMSC” [MeSH Terms] OR (“synovial mesenchymal stem cells” [All Fields] AND (“mesenchymal stem cells” [MeSH Terms] OR (“mesenchymal” [All Fields] AND “stem” [All Fields] AND “cells” [All Fields]) OR “mesenchymal stem cells” [All Fields]) AND “cartilage” [All Fields]) and “cartilage”, “Synovial Mesenchymal Stem Cells”. Screened studies were reviewed by title and/or abstract to determine study eligibility based on inclusion criteria. In cases of disagreement, a third reviewer made the final decision.

### 2.2 Inclusion criteria

This systematic review included randomized controlled trials (RCTs), clinical studies, and animal experiments published between 2004 and 2025. The inclusion criteria required: (1) complete English-language full-text availability; (2) studies from all geographic regions; and (3) human participants consisting of adult patients (≥18 years) of both sexes with radiographically confirmed knee osteoarthritis (Kellgren-Lawrence [KL] grade 2–3).

### 2.3 Selection and data collection

Article screening was performed in two phases according to the predefined selection criteria. First, titles and abstracts were evaluated for preliminary eligibility. Subsequently, full-text review was conducted for final inclusion confirmation. The screening process included: (1) removal of duplicate publications through systematic deduplication; (2) verification of strict adherence to search criteria; and (3) quality control through independent dual evaluation by two investigators (J.M.S. and Y.S.Q.). Any discrepancies were resolved through adjudication by a senior researcher (Y.S.X.).

### 2.4 Quality assessment and risk of bias

The risk of bias was assessed using the Systematic Review Center for Laboratory Animal Experimentation (SYRCLE) tool, which is specifically designed for animal studies (Hooijmans et al., 2014). This tool evaluates bias across several domains: Random sequence generation (selection bias), allocation concealment (selection bias), blinding of participants and personnel (performance bias), blinding of outcome assessment (detection bias), incomplete outcome data (atrition bias), selective reporting (reporting bias), and other sources of bias.

## 3 Results

### 3.1 Literature search and study identification

**FIGURE 3:**
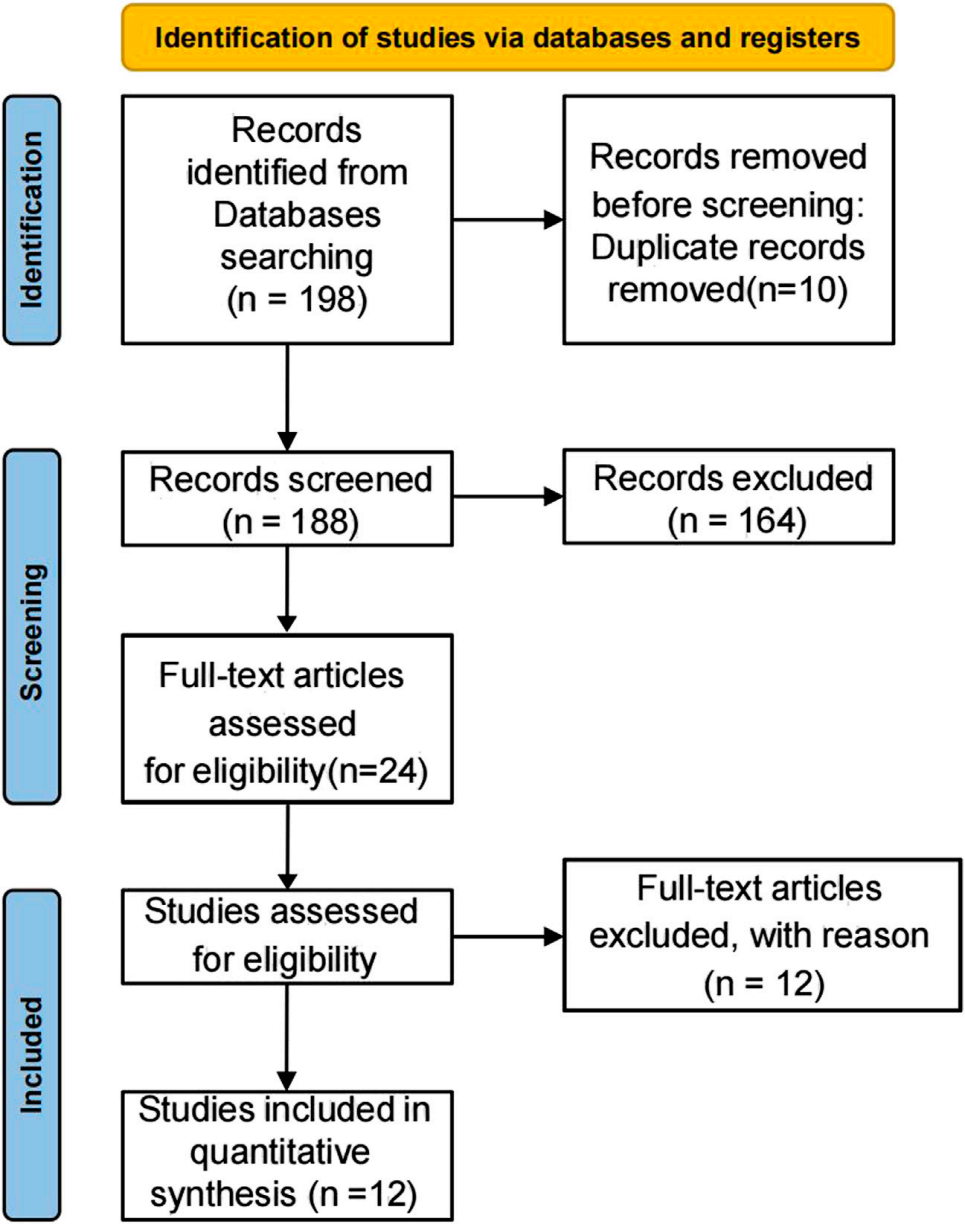
Literature screening process.

The initial literature search identified 198 potentially relevant studies. After removing 7 duplicate publications, 183 records remained for screening. Title and abstract review excluded 164 irrelevant studies. Full-text assessment was performed on the remaining 19 articles, of which 12 ultimately qualified for inclusion. The complete study selection process is detailed in the PRISMA flowchart (Figure 3; Table 1).

**TABLE 1:** Summary of characteristics of animal models.

Author, year	Animal	Age	Sample size	Gender	Weight	Animal model	Method of induction
Sun et al. (2023)	Rats	10 weeks	40	Male	NR	Knee osteoarthritis	Chemical; mono-iodoacetate injection
Long et al. (2023)	Mice	NR	88	NR	NR	Knee osteoarthritis	Surgical; resection of the medial meniscus
Xu et al. (2021)	Rats	NR	40	Male	200–220 g	Knee osteoarthritis	Surgical; resection of the medial meniscus
Gao et al. (2024)	Rats	NR	36	NR	NR	Knee osteoarthritis	Surgical; resection of the medial meniscus
Tao et al. (2017)	Rats	12 weeks	NR	Male	300–350 g	Knee osteoarthritis	Surgical; resection of the medial meniscus
Wang et al. (2021)	Mice	NR	20	NR	NR	Knee osteoarthritis	Physics; cold water stimulation
Lu et al. (2021)	Rats	NR	36	Male	300–350 g	Knee osteoarthritis	Surgical; resection of the medial meniscus
Zhao et al. (2024)	NR	NR	NR	NR	NR	NR	NR
Zheng et al. (2022)	NR	NR	NR	NR	NR	NR	NR
Kong et al. (2023)	NR	NR	NR	NR	NR	NR	NR
Zeng et al. (2022)	Mice	8 weeks	48	Male	NR	Knee osteoarthritis	Chemical; mono-iodoacetate injection
Qiu et al. (2021)	NR	NR	NR	NR	NR	NR	NR

### 3.2 Risk of bias

**FIGURE 4:**
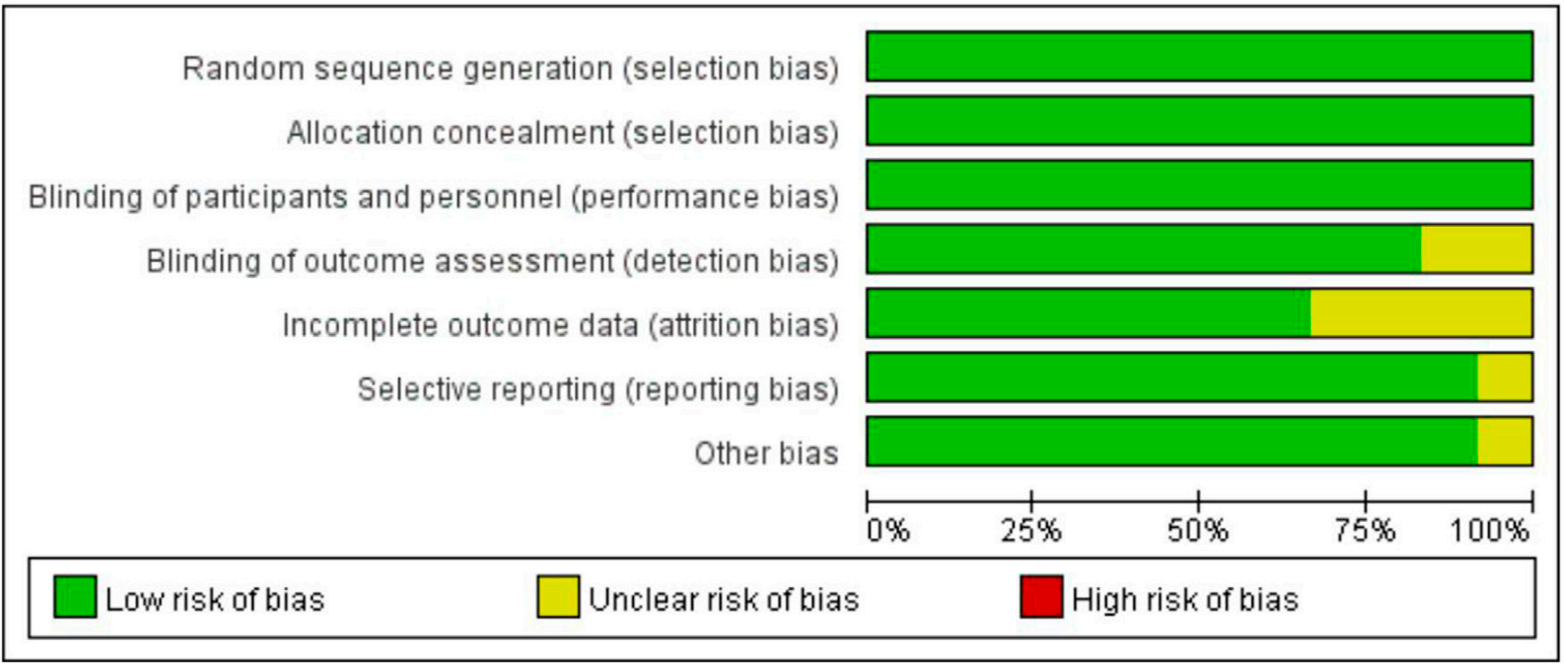
The graph of risk assessment. Risk of bias graph about KOA treated with SMSCs-Exos.

The overall and individual study results of the SYRCLE bias risk assessment are detailed in Figure 4. Overall, the risk of bias in most of the eligible studies was unclear. Some papers mentioned that animal groups were randomly assigned, but did not provide specific descriptions of the randomization procedure and allocation concealment methods. All literature showed that the baseline characteristics of the intervention and control groups were comparable at the beginning of the experiment. Some studies have not established animal models for *in vivo* studies. No selective reporting or other sources of bias were identified in any of the studies.

### 3.3 Animal model

The SMSCs in the twelve studies were derived from human (n = 6), Sprague Dawley mouse (n = 4) and did not mention a clear method for producing SMSCs (n = 2). Three studies established mice models, four with no animal model, and the remaining five with rat models. In all animal studies, one study used cold water stimulation to induce KOA in mice, while five studies’ established an OA model by completely transecting the medial collateral ligament and the medial meniscus, by cutting the meniscus at the narrowest point without damaging the tibial surface, and transecting the anterior cruciate ligament (Table 1).

### 3.4 Identification of SMSCs and their exos

Regarding the extraction method used for obtaining SMSCs, five studies used collagenase, one study used trypsin to isolate SMSCs, two studies purchased the finished SMSCs, and three studies did not describe the method of extracting SMSCs. In the 12 studies, multiple methods were used to confirm the success of SMSCs extraction, including microscopic observation of the spindle-shaped shape of extracted SMSCs (n = 10), and detection of SMSCs-specific surface markers by flow cytometry (n = 8). The osteogenic ability of SMSCs is identified by alizarin red staining, the lipid-forming ability of SMSCs is identified by oil red O staining, and the chondrogenic ability of SMSCs is identified by Alcian blue staining (n = 6) (Table 2).

**TABLE 2:** Characterization of MSCs and their derived secretome and EVs *in vitro*.

Author, year	Source	Isolation method	Characterization	Size distribution	Marker expression
Sun et al. (2023)	Mouse	Supernatants after centrifugation	TEM, Western blot, NTA	50–160 nm	CD63^+^, CD81^+^, CD9^+^
Long et al. (2023)	Mouse	Centrifugation, adherence to tissue culture flask, Supernatants after centrifugation	Flow cytometry, TEM, Western blot, NTA	30–100 nm	CD90^+^, CD105+,CD34-, CD45-, CD63^+^, HSP70+, Calnexin-
Xu et al. (2021)	Human	Centrifugation, adherence to tissue culture flask, Supernatants after centrifugation	Flow cytometry, TEM, Western blot, NTA, Osteogenesis, chondrogenesis and adipogenesis	NR	CD73^+^, CD90, CD105+, CD11b, CD19, CD63^+^, CD81^+^, Tsg101-
Gao et al. (2024)	Human	Centrifugation, adherence to tissue culture flask, Supernatants after centrifugation	Chondrogenesis	NA	CD44^+^, CD90^+^, CD105+
Tao et al. (2017)	NR	Centrifugation, adherence to tissue culture flask, Supernatants after centrifugation	Flow cytometry, TEM, Western blot, NTA, Osteogenesis, chondrogenesis and adipogenesis	30–150 nm	CD44^+^, CD73^+^, CD90^+^, CD105+, CD151+, CD11b-, CD19-, CD34-, CD45-, CD133-, CD9^+^, CD63^+^, CD81^+^, Alix+
Wang et al. (2021)	Mouse	NR	TEM, Western blot, NTA	100–120 nm	CD63^+^, CD81^+^
Lu et al. (2021)	Human	Centrifugation, adherence to tissue culture flask, Supernatants after centrifugation	Flow cytometry, TEM, Western blot, NTA, Osteogenesis, chondrogenesis and adipogenesis	100–120 nm	CD90^+^, CD151+, CD45-, CD133-, CD63^+^, CD9^+^, Calnexin-
Zhao et al. (2024)	NR	NR	Flow cytometry, TEM, Western blot, NTA, Osteogenesis, chondrogenesis and adipogenesis	100 nm	CD44^+^, CD73^+^, CD34-, CD45-, CD81, TSG101
Zheng et al. (2022)	Human	Centrifugation, adherence to tissue culture flask, Supernatants after centrifugation	Flow cytometry, TEM, Western blot, NTA, Osteogenesis, chondrogenesis and adipogenesis	30–150 nm	CD44^+^, CD44^+^, CD73^+^, SCA-1+, CD34-, CD45-, CD9^+^, CD63^+^, Alix+
Kong et al. (2023)	Human	Centrifugation, adherence to tissue culture flask, Supernatants after centrifugation	Flow cytometry, TEM, Western blot, NTA	60–300 nm	CD44^+^, CD73^+^, CD90^+^, CD105+, CD34-, CD45-, CD9^+^, CD63^+^, CD81
Zeng et al. (2022)	Mouse	Centrifugation, adherence to tissue culture flask, Supernatants after centrifugation	Flow cytometry, TEM, Western blot, NTA, Osteogenesis, chondrogenesis and adipogenesis	100 nm	CD44^+^, CD73^+^, CD34-, CD45-, CD63^+^, TSG101
Qiu et al. (2021)	Human	Centrifugation, adherence to tissue culture flask, Supernatants after centrifugation	TEM, Western blot	NR	HSP70+, CD9^+^, CD81

There are numerous approaches to verify the expression of the exosomes under examination. Regarding the identification of exosomes derived from SMSC, eleven studies used Western blot to detect their surface markers and used electron microscope to observe the diameter of exosomes, whereas the remaining studies do not mention how to successfully test for exosome extraction (Table 3).

**TABLE 3:** Treatment parameters and analyses for included studies evaluating the efficacy of MSC-based therapies for OA treatment.

Author, year	Group	Study timepoints	Conc./Volume/Frequency	Method of analysis		
				Gross	Histology	IHC
Sun et al. (2023)	Control group, KOA group, exo group, BMP-7-exo group	8 weeks	1 × 10^11^ exosome particles/mL/100 μL/3 d	Gross morphology	HE, Safranin-O-Fast-Green	collagen II, aggrecan, iNOS, CD206
Long et al. (2023)	Control group, OA group, OA + PBS group, OA + SMSC-Exo group, OA + SMSC-oe-NC group, OA + SMSC-Exo-oe-Matn3 group, OA + SMSC-Exo-sh-NC group, OA + Exo-sh-Matn3 group	4 weeks	100 μg/L SMSC-Exo or 1 × 10^11^ lentivirus/10 μL/1 w	Gross morphology	HE, Safranin-O-Fast-Green, Toluidine blue	Matn3
Xu et al. (2021)	Control group, OA + PBS group, OA + Exo group, OA + SMSCs group, OA + KGN group, OA + Exo/KGN group, OA + Exo/KGN + SMSCs group, OA + E7-Exo/KGN + SMSCs group	5 weeks	1000 μM/L/100 μL/1 w	Gross morphology	HE, toluidine blue, safranine green	Collagen Ⅰ, collagen Ⅱ
Gao et al. (2024)	Sham group、OA group、OA+ SMSC-pcDNA3.1 group, OA+ SMSC-pcDNA3.1-MALAT1 group, OA+ SMSC-NC group, OA+ SMSC-sh-MALAT group	NR	NR	Gross morphology	Safranin-O and fast green	Col2a1, Sox 9, ACAN
Tao et al. (2017)	Normal group, OA Group, OA + SMSC-Exos Group, OA + SMSC-140-Exos group	12 weeks	1 × 10^11^ exosome particles/mL, 100 μL, 1 w	Gross morphology	Safranin-O and fast green	Collagen Ⅰ, collagen Ⅱ
Wang et al. (2021)	Normal group, OA Group, OA+ SMSC-Exo group, OA+ SMSC-155-5p-Exo group	2 weeks	1 × 10^11^ exosome particles/mL, 30 μL, 1 w	Gross morphology	NR	collagen Ⅱ, apoptotic proteins, P65
Lu et al. (2021)	sham group OA group, OA+ GW group, OA+ EVs group, OA + EV-NC group, OA+ EV-inhibitor group	28 days	1 × 10^11^ exosome particles/mL, 30 μL, 1 w	Gross morphology	HE, Safranin-O and fast green	apoptotic proteins
Zhao et al. (2024)	NA	NA	NA	NA	NA	NA
Zheng et al. (2022)	NA	NA	NA	NA	NA	NA
Kong et al. (2023)	NA	NA	NA	NA	NA	NA
Zeng et al. (2022)	OA group, EVs group, EV + LV-NC group, EV + LV-LRP12 group	4 weeks	20 μg EV	Gross morphology	Safranin-O and fast green	collagen Ⅱ
Qiu et al. (2021)	NA	NA	NA	NA	NA	NA

### 3.5 Evaluation of the repair effect of SMSC-derived exosomes on chondrocytes

Twelve studies investigated the anti-inflammatory effects of SMSCs-derived exosomes by establishing IL-1βOA models *in vitro*. Eight studies demonstrated that SMSC-derived exosomes might inhibit apoptosis and promote cell proliferation in OA animal models by scoring them directly. Seven studies demonstrated that SMSC-derived exosomes could reduce proteins such as proteoglycan and reverse the course of OA. All studies in this systematic review have proved that SMSC-derived exosomes can induce chondrocyte proliferation and reduce chondrocyte matrix secretion, which protect against KOA. However, different exosomes contain different miRNA, which have different protective effects on cartilage (Table 4).

**TABLE 4:** Summary of key outcomes for included studies evaluating MSC-based therapies for OA treatment.

Author, year	Key outcomes (*in vitro*)	Key outcomes (*in vivo*)
Sun et al. (2023)	BMP-7-exos reversed the inhibition of LPS on RAW264.7 and chondrocytes, promoted the polarization of RAW to M2, and inhibited the polarization of M1. BMP-7-exo inhibited apoptosis of chondrocytes by macrophage M2 polarization	BMP-7-exo had a significant anti-inflammatory effect on KOA by shifting macrophage polarization from M1 to M2 phenotype
Long et al. (2023)	SMSC-Exo can play a role in OA by delivering MATN3 to the chondrocytes. MATN3 inhibits the release of downstream inflammatory cytokines, ECM degradation, and autophagy defects by interacting with IL-17A. MATN3 inhibits IL-17A-induced activation of the PI3K/AKT/mTOR signaling axis, thereby inhibiting IL-1β-induced ECM degradation and autophagy defects in chondrocytes	MATN3 inhibits the release of inflammatory cytokines, ECM degradation, and autophagy defects by interacting with IL-17A
Xu et al. (2021)	Exosome-mediated delivery of KGN for SF-MSC chondrogenic differentiation *in vitro*. E7-Exo-encapsulation effectively circumvents the solubility problem of KGN, alleviates the aggregation of KGN inside cells, and induces a stronger differentiation of SF-MSCs to chondrocytes	Transplantation of MSCs loaded with E7-Exo/KGN showed superior efficacy in reverting the damaged cartilage to the normal state in the DMM model, whereas exosomes alone MSC alone, or MSCs loaded with non-targeting exosome/KGN could only lead to partial regeneration
Gao et al. (2024)	miR-212-5p mimics obviously inhibited MYD88 expression compared to the mimics group, while miR-212-5p inhibitor promoted MYD88 expression	Decreased MALAT1 induced chondrogenic differentiation of hSMSCs. Inhibition of MALAT1 promoted the expression of Sox9, ACAN, and Col2a1 suggesting SMSC-sh-MALAT1 prevents OA in rats
Tao et al. (2017)	SMSC-Exos activated YAP, decreased ECM secretion of articular chondrocytes, and induced proliferation and migration of articular chondrocytes. SMSC-Exos decreased ECM secretion and induced proliferation and migration of articular chondrocytes through activation of YAP. Exosomes derived from SMSC-140-Exos induced proliferation and migration of AC without decreasing ECM secretion	SMSC-140-Exos slowed the progression of early OA and prevented the severe damage to knee articular cartilage in the OA model caused by instability of the knee joint
Wang et al. (2021)	The SMSC-Exos promote proliferation and migration but inhibit apoptosis in osteoarthritic chondrocytes. Exosomal miR-155-5p increases proliferation, migration, and ECM secretion and attenuates apoptosis in osteoarthritic chondrocytes	Exos with or without some special modifications may have an important role in clinical applications
Lu et al. (2021)	SMSC-EVs suppress IL-1β-induced chondrocyte apoptosis and inflammation. SMSC-EVs transmit miR-26a-5p into SW1353 cells to upregulate miR-26a-5p expression. SMSC-EVs ameliorate IL-1β-induced chondrocyte apoptosis and inflammation by carrying miR-26a-5p	EVs exert protective effects against cartilage damage in OA by carrying miR-26a-5p to target PTEN expression
Zhao et al. (2024)	miR-485-3p was transferred by Exos, which might affect cartilage injury *in vitro*. Exos attenuated cartilage injury by modulating miR-485-3p expression	NA
Zheng et al. (2022)	SMSC-212-5p-Exos) inhibit IL-1β induced ELF3 expression in chondrocytes. SMSC-212-5p-Exos attenuate IL-1β induced chondrocyte degeneration and degradation. SMSC-212-5p-Exos attenuate IL-1β induced inflammatory responses in chondrocytes	NA
Kong et al. (2023)	SMSC and its derived exosomes enhanced chondrogenesis. MiR-320c promoted chondrogenesis through targeting ADAM19	NA
Zeng et al. (2022)	EVs alleviate IL-1b-induced growth inhibition, ECM degradation and inflammation in chondrocytes. Overexpression of LRP12 activates the AKT/b-catenin signaling pathway and blocks the alleviating roles of EVs in chondrocytes	NA
Qiu et al. (2021)	miR-129-5p may achieve a decrease in the inflammatory response and cell apoptosis by inhibiting HMGB1. Inhibiting miR-129-5p in HS-MSC-Exo aggravated IL-1β-mediated chondrocyte injury	NA

All the studies included in the review carried out *in vitro* cell experiments, and all proved that SMSC-derived exosomes could effectively improve IL-1β-induced cartilage damage, increase the proliferation of chondrocytes and cartilage tissue repair, and some specific functional mirnas could also increase the secretion of extracellular matrix. All studies including animal experiments have also proved that intra-articular injection of exosomes can effectively alleviate cartilage damage after modeling, and the degree of cartilage damage was graded by OARSI score, and the tissue was stained with HE and Safranin O and fast green. It was found that exosomes had a good effect on delaying the progress of KOA, especially the therapeutic effect of some specific mirnas was better than that of common mirnas, and the tissue staining results and OARSI scores were closer to the blank control group.

Sun et al. (2023) by preparation of BMP-7 overexpression exosomes, *in vitro* cell experiments showed that BMP-7 overexpression exosomes had a better effect on cartilage repair, and could significantly inhibit the LPS-induced polarization of RAW264.7 to M1 and to M2 direction. It was further demonstrated that polarization of macrophages to M2 promoted chondrocyte proliferation and migration and inhibited chondrocyte apoptosis. *In vivo* experiments have also proved that BMP-7-exos can inhibit the degeneration of articular cartilage and slow down the progress of KOA, and can induce the polarization of macrophages in synovial tissue to M2 direction, which plays an anti-inflammatory role.

Long et al. (2023) through bioinformatics analysis, we found that SMSC-Exo could transmit MATN3 and regulate IL-17 signaling pathway to participate in the development of OA, which was used as the entry point for further research. Firstly, the DMM animal model was established. The results of PCR and tissue staining showed that MATN3 overexpression could effectively alleviate cartilage damage. Moreover, overexpression of MATN3 inhibited IL-1β-induced extracellular matrix degradation and autophagy defects in chondrocytes *in vitro*. Further studies then targeted MATN3 and found that MATN3 inhibited IL-17A-induced activation of PI3K/AKT/mTOR signaling axis, thereby inhibiting IL-1β-induced extracellular matrix degradation and chondrocytes autophagy defects.

Xu et al. (2021) promoted the differentiation of SMSC into chondrocytes and reduced chondrocyte damage *in vitro* by exosome-loaded Kartogenin (KGN). The exosome envelope provides protection for KGN, and KGN can promote the differentiation of SMSC into chondrocytes, achieving a two-way promotion effect. On the basis of the cell experiments, the team further established an animal model for study, and found that intra-articular injection of KGN-loaded exosomes could continue to play a role in the joint cavity. Further studies showed that the cartilage defects caused by the DMM model were repaired with KGN-loaded exosomes, which were closer to the normal cartilage morphology.

Gao et al. (2024) compared the time of induced differentiation of mesenchymal stem cells and found that the expression of MALAT1 decreased significantly, so they used it as a research entry point for in-depth study. Bioinformatics studies showed that mi-212-5p could significantly inhibit the expression of MALAT1 and play a role in inducing SMSC chondrogenesis. Further *in vivo* animal studies showed that inhibition of MALAT1 greatly promoted the differentiation of SMSCS into chondrocytes and delayed the progression of arthritis.

Tao et al. (2017) used qPCR to confirm that SMSC-Exo promoted the proliferation and migration of chondrocytes by activating Hippo/yes-associated protein (YAP) pathway. In this study, the expression level of miRNA was measured by microarray, miR-140-5p is the highest expression level selected for the study. The team consulted relevant data and proposed that RalA might be the target of its action, which was verified by detecting the expression of SOX9 and Aggrecan. Finally, RalA was identified as the target of miR-140-5p. Animal studies also revealed similar results to *in vitro* studies. Compared with the OA group of rats, the SMSC-140-Exos group rats had less cartilage wear, and OARSI score, and were more akin to the normal group. This study strongly demonstrates that miR-140-5p can significantly slow disease progression in the early stage of OA.

In their study, Wang et al. (2021) formed miRNA profiling of synovial tissues from both healthy controls and KOA patients, revealing 52 differentially expressed miRNAs. Among these, the top 20 most significantly altered miRNAs were subjected to RT-qPCR validation, which subsequently identified four particularly prominent miRNAs. Notably, miR-155-5p emerged as the most abundant miRNA in SMSC-Exos. Western blot analysis further revealed that miR-155-5p mediates dual regulatory functions, significantly inhibiting cellular apoptosis while promoting extracellular matrix (ECM) secretion. Bioinformatics analysis using MiBase and other prediction tools identified Runx2 as a putative target of miR-155-5p. Subsequent experimental validation through three complementary approaches - quantitative gene expression analysis, dual-luciferase reporter assay, and Western blotting - conclusively demonstrated Runx2 as a direct target of miR-155-5p. Notably, Runx2 demonstrates significantly higher expression in osteoarthritic synovial tissue compared to normal tissue. Functional studies revealed that Runx2 overexpression counteracts the anti-apoptotic effects of miR-155-5p in chondrocytes, as confirmed through comprehensive assessment using flow cytometry, Western blot analysis, and CCK-8 assays. In KOA chondrocyte models, we observed elevated Caspase-3 activity, which was markedly reduced following miR-155-5p treatment (SMSC-155-p group). Consistent with these *in vitro* findings, rat femoral condyle cartilage histology and OARSI scoring in *ex vivo* experiments corroborated the therapeutic potential of miR-155-5p. Collectively, these results provide compelling evidence that miR-155-5p exerts chondroprotective effects capable of mitigating osteoarthritis progression, highlighting its significant clinical translation value.

Lu et al. (2021) proved that exosomes contained miR-26a-5p to SW1353 cells through a series of cell experiments, inhibiting cell apoptosis and inflammation induced by IL-1β. To determine the downstream mechanism of miR-26a-5p, the researcher searched multiple databases and did genetic testing on them, and finally discovered PTEN as its binding site. PTEN has opposite effects on exosomes on chondrocytes. This study included *in vivo* and animal experiments, which proved that Exos targets PTEN expression by miR-26a-5p, and demonstrated the therapeutic effect of miR-26-5p on OA.

Mingjun Qiu et al. (Zhao et al., 2024) investigated using a miRNA microarray experiment to investigate Exo-induced differently expressed miRNAs, focusing on miR-485-3P, the microRNA with the highest expression level in Exos. The authors searched StarBase and the remaining four databases, predicated five potential target genes for miR-485-3P. The study was verified using an IL-1β-induced OA cell model, which confirmed that NRP-1 is the target gene of miR-484-3P, and NRP-1 is negatively associated with miR-485-3P. After the silence and overexpression of NRP-1 activate and inhibit of PI3K/Akt pathway, Aggrecan, Collagen II, and matrix metalloproteinases (MMP) 13 in chondrocytes were detected. It was finally confirmed that miR-484-3P carried by Exos activates NRP-1 and protects cartilage through the PI3K/Akt pathway.

Zheng et al. (2022) identified a significant downregulation of miR-212-5p accompanied by concurrent upregulation of ELF3 expression in OA patient synovial tissues. Their investigation revealed that miR-212-5p overexpression suppressed IL-1β levels in chondrocytes, while ELF3 expression showed a positive correlation with IL-1β production. Through bioinformatics analysis using TargetScan and miRBase databases combined with experimental validation, the researchers established ELF3 as a direct downstream target of miR-212-5p in SMSCs. Importantly, their findings demonstrate that miR-212-5p exerts protective effects by attenuating IL-1β-induced ELF3 expression, thereby mitigating chondrocyte degeneration and reducing inflammatory factor production.

Kong et al. (2023) demonstrated that treatment with SMSC-Exo, SMSC-NC-Exo, and SMSC-miR320c-Exo significantly attenuated cartilage damage while enhancing chondrocyte proliferation in OA models. These treatments markedly downregulated pro-inflammatory cytokines (IL-1β, IL-6, and TNF-α) and apoptosis-related markers (Bcl2 and Caspase3), indicating both anti-inflammatory and anti-apoptotic effects. Particularly, SMSC-derived exosomal miR-320c showed pronounced efficacy in preventing cartilage ECM degradation and chondrocyte apoptosis. Furthermore, mechanistic studies revealed that miR-320c-containing exosomes promoted chondrogenesis through targeted inhibition of ADAM19 expression.

Zeng et al. (2022) found that Exos derived from SMSC could effectively reduce IL-1β-induced extracellular matrix degradation and inflammatory factors such as IL-6 in cells. Then using mRNA microarray techniques, ten of the highest miRNA expression differences were screened, and the largest miR - 130 - b - 3 p from the OA model and control group was chosen as the research object. In order to predict the target genes of miR-130b-3p, the study searched 5 databases such as StarBase, and 5 potential genes were ultimately selected. Using RT-qPCR to detect the expression of these genes, only LRP12 was significantly reduced, which was identified as the target gene of miR-130b-3p. Finally, *in vivo* studies were conducted to confirm the experimental results via establishing animal experimental models. Animal experiments showed that miR-130b-3p could improve the levels of inflammatory factors such as IL-6 *in vivo*, reduce the wear of chondrocytes, and significantly repair cartilage damage, as confirmed by the OARSI. This study demonstrated that miR-103b-3p can effectively repair cartilage damage caused by OA *in vitro* and *in vivo*, which has great development prospects.

Qiu et al. (2021) conducted *in vitro* experiments on OA chondrocytes finding the cell COX2 and MMP13 expression of inflammatory markers to reflect the degree of inflammation of OA chondrocytes. In this study, by comparing the synovial fluid of KOA patients and healthy people, the expression level of miR-129-5p was the greatest difference, so it was determined as the research object. HMGB1 (box-1 with high mobility), a kind of regulation of gene transcription factor, can stimulate the inflammatory response (Steinle, 2020). At the same time, HMGB1 was also determined as a target gene of miR-129-5p in a variety of ways. In this study, the expression levels of inflammatory factors in OA chondrocytes were detected by silencing and overexpressing HMGB1 and miR-129-5p, so as to highlight the therapeutic effect of miR-129-5p on OA.

## 4 Discussion

OA, a prevalent degenerative joint disease worldwide, is characterized by progressive cartilage degeneration (He et al., 2022). The current therapeutic options for OA remain limited due to its complex and poorly understood pathogenesis, creating an urgent need for more effective treatment strategies to halt disease progression. Cell-free therapies utilizing exosomes have emerged as a promising approach for cartilage repair, leveraging their natural role as key mediators of intercellular communication through paracrine mechanisms (Wu et al., 2019). Particularly, exosomes derived from SMSCs demonstrate therapeutic potential by promoting chondrocyte regeneration and migration while reducing cartilage degradation in KOA. Notably, these exosomes modulate ECM secretion in chondrocytes. This systematic review comprehensively evaluates the clinical efficacy of various SMSC-derived exosomal microRNAs in cartilage repair.

In the above 12 studies, different types of exosomes were investigated to evaluate the therapeutic effect after surgery and to determine the regeneration of cartilage tissue by *in vivo* experiments. All studies confirmed that exosomes derived from SMSCs were able to maintain and rebuild articular cartilage. The main functional mirnas in exosomes were found and verified by isolation and extraction. Studies have shown that these mirnas not only maintain and reestablish chondrocyte function, but also promote the secretion of extracellular matrix by chondrocytes (Figure 1).

In the 12 included studies, it was confirmed that SMSC-derived exosomes could maintain and promote cartilage repair and reduce the degree of cartilage damage by *in vitro* cell experiments. By isolating and extracting the main functional mirnas, it was found that these functional mirnas had a good therapeutic effect on cartilage injury. Different sources of SMSCs have been used in each study, among which human-derived SMSCS have been the most studied, followed by mouse-derived SMSCs, and there are also SMSCs directly purchased and successfully extracted. The extraction methods of exosomes are also different, but most studies use ultracentrifugation to extract exosomes. Although the methods of each study were different, there was no statistically significant difference in the final evaluation indicators, as detailed in Table 2.

Among all the included studies, 5 studies did not establish animal models for *in vivo* study, 2 studies established KOA models by intra-articular injection of mono-iodoacetate, 1 study established KOA models by cold water stimulation, and the rest established KOA models by surgery. Although the establishment methods of animal models are different, the indicators to evaluate the success of model establishment are similar, so it has little impact on the research outcome.

OA progression involves critical pathological changes in cartilage, notably ECM degradation and chondrocyte apoptosis (Fujii et al., 2022; Peng et al., 2021). The ECM serves as both a structural scaffold and a biochemical regulator, facilitating nutrient transport to chondrocytes while maintaining tissue homeostasis and mechanical integrity (Rahmati et al., 2017). Our findings demonstrate that SMSCs and their exosomes effectively downregulate MMP3 and MMP13—key matrix-degrading metalloproteinases—while upregulating collagen type II expression, the predominant structural component of healthy cartilage (Peng et al., 2021; Rahmati et al., 2017).

Multiple studies highlight the therapeutic potential of miRNAs in OA. Rasheed et al. (2016) identified that miR-26a-5p downregulates inducible nitric oxide synthase (iNOS) and restores chondrocyte homeostasis, thereby alleviating OA progression. This miRNA demonstrates consistent protective effects across arthritic conditions - in rheumatoid arthritis, activated miR-26a suppresses apoptosis and inflammation while stimulating chondrocyte proliferation (Jiang and Cao, 2020), whereas its downregulation in OA correlates with enhanced apoptosis, inflammatory damage, and synovial hyperplasia (Zhao et al., 2019). Parallel research reveals additional therapeutic miRNAs: mmu-miR320c-3p inhibits OA progression *in vivo* through β-catenin targeting (Hu et al., 2019). While miR-485-3p overexpression protects against osteoarthritic cartilage injury (Zhou et al., 2021).

Emerging evidence indicates that SMSC-derived exosomes mediate cartilage protection through targeted molecular delivery. These exosomes transport regulatory miRNAs (e.g., miR-150-5p and miR-212) that significantly downregulate MMPs, thereby promoting collagen type II accumulation while suppressing chondrocyte apoptosis (Zheng et al., 2022; Chen et al., 2018). Complementing these findings, Tao et al. (2017) demonstrated that exosomal components inhibit ECM secretion via SOX9 pathway suppression.

Recent advances in miRNA research have significantly enhanced our understanding of OA pathophysiology, sparking renewed interest in this field (Tsai et al., 2017; Mao et al., 2017). Synovial tissue exists only in a small number of parts of the human body. Therefore, there are relatively few studies that specifically explore exosomes derived from synovial mesenchymal stem cells in depth. However, there are more studies related to exosomes derived from umbilical cord blood mesenchymal stem cells, bone marrow mesenchymal stem cells and adipose-derived mesenchymal stem cells, and their applications in cartilage injury have also been deeply studied.

Previous studies Chang et al. (2025) have shown that exosomes secreted by HUCMSCs pretreated with PRP have a greater chondrogenic ability than those without PRP. The study also demonstrated that exosomes from the pretreatment group could suppress chondrocyte inflammation and restore cartilage matrix proteins without inhibiting matrix degradation. Related studies have also shown that subcutaneous fat (SC) stromal cells derived exosomes (MSCs^SC^-Exos) specifically encapsulates miR-199a-3p in chondrocytes and is delivered to the deep part of the knee joint by intra-articular injection. To achieve good therapeutic effect (Zhao et al., 2023). Exosomes derived from various types of cells have been widely used in cartilage injury, such as M2 macrophages (Zhang et al., 2025), human fetal cartilage stem cells (Lee et al., 2025), auricular chondrocytes (Kobatake et al., 2025), etc. Exosomes themselves can play a role in promoting cartilage repair, and they can also be used as a drug delivery medium to amplify their role in promoting cartilage repair (Wu et al., 2025; Tu et al., 2025).

In recent years, exosome-based therapies have shown significant advantages in the field of osteoarthritis treatment, especially in the treatment of KOA, which has shown unique safety and clinical transformation potential. As a kind of natural extracellular vesicles, exosomes have the following outstanding advantages: first, they are highly biocompatible and can be quickly removed by the human body through normal metabolic pathways after local therapeutic effects, thus avoiding possible side effects caused by long-term retention (Chang et al., 2018). Second, as compared with stem-cell therapy, exosomes are nonproliferative and completely avoid the risk of tumorigenesis, a property that offers a significant advantage in long-term safety (Amsar et al., 2022; Matthay, 2017). In addition, exosomes require significantly lower storage conditions than SMSC, which can be stably stored at 4°C for 72 h and frozen at −80°C for months, which greatly reduces the logistics and storage costs in clinical applications (Fang and Vangsness, 2024).

The existing research data show that exosomes can effectively regulate the inflammatory microenvironment in the joint cavity and promote the proliferation of chondrocytes and extracellular matrix synthesis by carrying bioactive substances such as specific mirnas, cytokines and growth factors (Li Z. et al., 2020). Animal studies have shown that intra-articular injection of exosomes can significantly improve the pain behavior score of KOA model animals, and histopathological analysis confirmed that it can effectively delay the process of cartilage degeneration, and no obvious local or systemic toxic reactions were observed. In terms of immunogenicity, homologous exosomes show a very low risk of immune rejection, which provides an important guarantee for their clinical transformation.

In terms of the indications of exosomes for the treatment of cartilage damage caused by KOA, most studies believe that MSC-Exos has a better therapeutic effect on KOA patients in the early and middle stage (KL 1–2). Grade 1–2 KOA is mainly characterized by mild cartilage damage and local inflammation, and the joint microenvironment is not completely unbalanced (Mao et al., 2018). Exosomes can inhibit inflammation (IL-1β, TNF-α), promote chondrocyte proliferation and matrix synthesis (collagen type Ⅱ, proteoglycan) by delivery of miRNA (such as miR-140-5p, miR-92a-3p), and delay the progress (Vizoso et al., 2019). For grade 3–4 KOA (large cartilage defect, bone sclerosis, or deformity), SMSC-Exos has limited repair ability. Advanced lesions are accompanied by hypoxia, fibrosis, and abnormal mechanical stress, which may inhibit the targeted delivery of exosomes and cellular responses (Vizoso et al., 2019). Irreversible changes in knee joint structure: exosomes are unable to directly repair osteophytes or reconstruct severely worn cartilage layers, but may reduce pain and inflammation by regulating the remaining subchondral bone to talk to the synovium (Wu et al., 2024).

The effects of exosomes on the mechanics and biological environment of the knee joint are mainly reflected in the regulation of the local microenvironment. The core mechanism of SMSC-Exos is to improve the biological microenvironment of joints, inhibit the polarization of M1 macrophages, and reduce the release of inflammatory mediators (Lai et al., 2025). Although exosomes cannot directly correct structural alignment abnormalities such as varus or valgus deformities, they may indirectly optimize mechanical loading by repairing cartilage, reducing local stress concentration in the knee, and relieving knee pain.

Based on the current research results, we suggest to accelerate the clinical transformation research of exosomes in the treatment of KOA. The specific research directions should include: (1) optimizing the separation and purification process of exosomes and establishing standardized production practices; (2) Conduct dose exploration and administration regimen optimization studies; (3) establish a long-term safety evaluation system; (4) explore exosome engineering modification strategies to enhance targeting and therapeutic effect. Through systematic preclinical and clinical studies, exosome therapy is expected to become a breakthrough therapy in the field of KOA treatment, providing a new treatment option for delaying disease progression and improving the quality of life of patients. The development of this innovative treatment strategy will greatly promote the progress in the field of osteoarthritis treatment, which has important clinical value and social significance.

The limitations of this study are mainly reflected in the following aspects. (1) The number of included studies was small. Because synovial tissue only exists in a relatively small number of parts, such as knee joints and shoulder joints, the number of studies on synovial tissue is small. (2) the heterogeneity of SMSCs and exosomes extraction methods. At present, MSC as one of the methods for the treatment of KOA has not been fully accepted by the law and is still in the exploratory stage. Therefore, there is a lack of standardized procedures for the extraction of SMSC and exosomes. (3) All the included studies only involved the efficacy of exosomes and did not compare with other treatment methods. As a new type of cell-free therapy, exosomes have a great improvement in safety and preservation compared with MSC, but they have not yet become an effective treatment method recognized by the public. Therefore, studies on the comparative efficacy of exosomes are still insufficient. In the future, researchers can compare the effect of exosomes with traditional therapies. To prove the effectiveness of exosomes in the treatment of KOA. (4) Lack of clinical research data. As exosomes have not been used in clinical practice on a large scale, there is still a lack of clinical data on exosomes. (5) Lack of long-term experimental data. The longest time of animal models established in each study was not more than 12 weeks, and there was a lack of long-term study data for comprehensive evaluation.

## Data Availability

The metadata and code used in this study can be obtained by contacting the corresponding author of this study.
